# Advanced integration of BIM and VR in the built environment: Enhancing sustainability and resilience in urban development

**DOI:** 10.1016/j.heliyon.2025.e42558

**Published:** 2025-02-08

**Authors:** Ali Shehadeh, Odey Alshboul, Madhar M. Taamneh, Aiman Q. Jaradat, Ahmad H. Alomari, Mai Arar

**Affiliations:** a Department of Civil Engineering, Hijjawi Faculty for Engineering Technology, Yarmouk University, P.O. Box 566, Irbid, 21163, Jordan; b Department of Civil Engineering, Faculty of Engineering, The Hashemite University, P.O. Box 330127, Zarqa, 13133, Jordan; c Department of Architecture, Faculty of Engineering, Al Al-Bayt University, Mafraq, Jordan

**Keywords:** Building information modeling, Virtual reality, Built environment, Sustainable urban development, Urban resilience

## Abstract

This study explores the strategic integration of Building Information Modeling (BIM) and Virtual Reality (VR) within the built environment, addressing the growing complexities of urban development. Through a detailed examination of BIM applications, the research highlights a 37 % reduction in design conflicts and iterations, thereby enhancing design precision and efficiency in urban construction projects. Simultaneously, VR technology is demonstrated to increase stakeholder engagement by 62 % and improve spatial awareness by 48 %, fostering greater community participation and inclusivity in the development process. The combined use of BIM and VR optimizes not only construction workflows but also considerably enhances environmental and socio-economic outcomes, such as a remarkable 20 % reduction in greenhouse gas emissions. It will, therefore, support advanced urban infrastructure development and provide a replicable model for sustainable and resilient urban planning. This research underlines the crucial contribution of integrating BIM and VR technologies to the development of sustainability and resilience of the built environment.

## 1 Introduction

The focal point of a variety of global challenges encompasses urban cities, which face various challenges ranging from climate change, resources management, and social cohesion [1]. Growing claims on infrastructure, plus pressing climate demands, call for innovative ways to plan and develop urban cities [2]. The "Climate for Cities (C4C) in the Southern Neighbourhood" is, perhaps, the first ever leading-edge initiative to transform downtown Irbid into a model of living in a sustainable and resilient city. The current project presents a fusion of two leading-edge technologies: Building Information Modelling (BIM) and immersive Virtual Reality (VR), both widely acclaimed over the last years, especially for their great capacity to change the AEC sectors beyond recognition.

BIM has a digital way of giving representation to physical and functional characteristics of places that increase the bar for raising better standards in the building's design and construction documentation processes [3]. It permits integrated project management that is capable of showing collisions at an early stage, which has its possibilities of reducing cost and time overruns [4]. While doing so, VR proposes far more interactive experiences that have tremendous value during the design process of preliminary ideas and space understandings. The integration of technology into urban development projects provides increased stakeholders' participation in design decisions and an effective means of communication for parties [5].

Urban cities are at the center of global challenges, dealing with issues like climate change, resource management, and social cohesion. These challenges require new and innovative ways to plan and develop cities to improve their infrastructure and sustainability. The "Climate for Cities (C4C) in the Southern Neighbourhood" initiative aims to turn downtown Irbid into a model of sustainable and resilient urban living. This project uses BIM and VR, both of which have greatly improved the architecture, engineering, and construction sectors by enhancing design accuracy and project management.

Despite the recognized potential for BIM and VR, their application in the urban design context of large-scale urban transformation projects is still considerably low, especially in developing countries [6]. Therein, the principal challenges entail the difficulty in integrating the stated technology into an already developed area, heavy financing investment being required, and requiring an encompassing participation of stakeholders: acceptance of which must flow from them [7,8]. Besides, the actual value of these technologies in realizing sustainability and resilience in urban projects has not been studied in terms of both quantity and quality [[9], [10], [11], [12], [13]]. This research tries to fill these gaps by demonstrating how BIM and VR can be effectively integrated to achieve the objectives of the C4C initiative in Irbid.

Beyond urban planning, BIM and VR can be applied to many other sectors, such as infrastructure maintenance, healthcare, and education. In infrastructure, BIM helps manage and maintain assets more effectively through enhanced data visualization and predictive maintenance. In healthcare, VR introduces new ways to treat patients and train medical staff, offering simulations that improve surgical accuracy and patient care. In education, VR's immersive environments can transform traditional teaching methods, providing simulations that mimic real-world scenarios. Similarly, BIM helps manage campus facilities and safety systems efficiently. These examples show the wide potential of BIM and VR to aid sustainable development in various fields.

This research aims to improve understanding of how BIM and VR are practically used in large urban redevelopment projects. While there has been some theoretical discussion and use in small projects, there is a lack of comprehensive studies on their use in larger, more complex urban settings, particularly those facing significant socio-economic issues. This limits our ability to apply these findings more broadly. This study seeks to provide solid evidence and a clear methodology for using these technologies in complex urban redevelopment efforts.

## 2 Literature review

This section provides an overview of the available literature and related research work regarding integrating BIM and VR applications within an urban development framework as an interesting area that is earning rapid popularity in urban planning, design, and management due to rapid technological advancements on both fields. This paper therefore presents a contextualizing basis on how BIM and VR may apply in various urban redevelopment projects, considering a review of related literature, identifying each technology's capability as well as their limits, thereby identifying the gap that this study is expected to fill.

This is further supported by the fact that recognition of the impact of climate change on our cities has led to the recognition of urban design and planning as a critical strategy in developing future resilience [6]. Multi-disciplinary integration in these fields is visibly lacking, further affecting the achievement of various sustainable development goals [42]. Moreover, the execution and operation of the urban changes by the tools of planning require the control of the appropriate territorial authorities to be certain that the planned changes are within the technical and legal regulations stipulated in the prevailing plans [14]. Therefore, the need for better urban and architecture design tools arises that may help mitigate the effects of climate change [15]. The most common is BIM technology [16]. BIM is a new type of information technology that is rapidly changing the AEC industry[17]. BIM enhances time, cost, coordination, communication, and quality [18]. The transformation under the building information model happens to be mainly beneficial in several design aspects, including tools, thinking, methods, and expressions [19]. Accordingly, BIM technology has started to see an increase in its application in design projects.

The research on building information systems in architectural building construction and education has been well researched. Enshassi et al. [20] researched and assessed the potential benefits of BIM in the architectural, engineering, and construction (AEC) industry. The results showed the four different elements or four-factor structures of BIM benefits. These elements are: (I) control over life cycle costs and environmental issues, (ii) facilitation of an effective building process, (iii) enhancement in design and quality, and (iv) support for decision-making. Kushwaha [21] concluded that BIM resolved the issues faced by the AEC industry and successfully mitigated the challenges involved in its adoption. While Apollonio et al. [22] have pointed out the manifold advantages of using BIM for the documentation and preservation of Architectural Heritage.

Moreover, on the basis of the established relationship between BIM and the process of understanding architectural design, much research has been carried out [[23], [24], [25], [26]]. In relation to BIM, it has been implemented in focusing on small-scale projects without minding urban growth [6]. Despite the advantages that were apparently identified, numerous BIM projects have scant limits. The lack of familiarity with BIM among professionals and stakeholders could affect design decisions [5]. For this reason, scholars started to research VR as an alternative that might improve the design process.

Recent research has demonstrated that the design of VR experiences is closely related to technological capabilities and limitations. Advanced technology and complex settings are needed for high-quality VR experiences to be created [27]. HMD is expected to reach an outstanding valuation of $25 billion by 2022 and reach a CAGR growth rate of 39.52 % from 2019 to 2025. Global Augmented Reality's 2019 data also reveal how much curiosity about immersive VR grew through the progress of improvement in technology relating to VR and decreasing prices of its cost [28,29]. Therefore, a wide range of unexplored possibilities is offered by VR, and it is used as a research tool [30].

Among them, the integration of VR and BIM in design has become one of the research focuses. This is because such an immersive experience is necessary to improve the interaction between architects and designers and their digital models. It helps them to create in a more intuitive creation and interaction process, as stated by [12]. Integration of VR with design and BIM has been the focus of studies. A study conducted by Zaker and Coloma [13] established how the integration of VR into workflows of BIM-enabled projects enhances collaboration and design review. With this approach, project participants are in a position to experience BIM models in the virtual environment, hence able to collaborate without necessarily having to be there physically. As indicated in this study, engagement of participants in more stimulating and interactive activities may be achieved through VR environments. This, in turn, fosters improved decision-making processes and collaboration across teams from different geographical locations.

Similarly, Alizadehsalehi et al. [9] applied 4D-BIM integrated into VR technology in construction planning in the light steel framing project by construction professionals. The results from this study imply that the uses of modern methods and technologies are likely to have potential benefits on construction. Lu et al. [10] did some research comparing the cost involved in constructing a park with VR to traditional ways. It also finds that the traditional way leads to a considerable raise in costs again found that VR and BIM platforms enable landscape architects to unleash their imagination and creativity easily [11].

Literatures have highlighted the transformative benefits gained when there is integration of BIM and VR technologies, especially in developing the city by [31,32]. Indeed, this paper finds out that greater integration of BIM and VR technologies into the industry would significantly facilitate an exponential application avenue for robotic assistants [33]. The literature search on the role of immersive technologies, BIM, and VR in sustainable smart cities revealed a fragmented yet dynamic field where trends and technological hotspots are fast-changing, emerging cyclically every 3–5 years [41].

In one such study, for example, multi-user VR integrated with BIM showcased a drastic improvement in communication, comprehension, and collaboration. Thereby, through review on a virtual platform, construction professionals can be capable of identifying design flaws and issues of constructability to be solved before physical productions begin [34,35]. Another study proposed BIM and VR-based intelligent systems for health risk assessment in the urban subway microenvironment and furthered urban environment management. The system gave, on one hand, a much clearer understanding of these kinds of risks to the experts and, on the other, made use of gamified simulations for greatly improving the passengers' ability [36].

Further, a new model was developed that combined the use of BIM with VR to optimize window-to-wall ratios and building orientation during the early design stages of a residential home. The study successfully reduced annual energy consumption through this approach and generally improved building energy performance [37]. It is these kinds of advances that show just what BIM and VR can achieve while transforming urban development and its sustainability. Integrating BIM with VR has the potential to expand the flexibility and efficiency of BIM. BIM allows one to create 3D models through which visualization of the prototype can be done, while in VR a person can actually experience the completed structure virtually [8]. This is also proposed that BIM integrated with VR technology will further improve the efficiency of workflow by attaining a better mutual understanding among the project participants [7]. Immersive experience helps the designers to make necessary edits towards design changes proposed by the user participant and adjusting any unexpected changes or failures in the early design phases [38]. Besides, the integration of this experience can facilitate equipping the architecture and engineering students with skills that are necessary [7].

Despite this, various challenges are yet to be overcome in architecture and urban development. Most research exploring the integration of BIM and VR focuses only on small-scale projects [6]. The least number of research studies focuses on the application of BIM and VR in urban development compared to their application in buildings and architectural education. Also, most of the projects do not contain complex attributes to deal with social and climatic issues [[9], [10], [11], [12], [13]]. Also, projects during the design stages are not taken into consideration in the studies. Consequently, this study tried to cover these existing gaps in a case study that contains a focus on social as well as the environmental matters. It examined the potential usage of BIM and VR technologies for enhancing the design process in the case study and reviewed the advantages and challenges associated with using this technology in urban development.

## 3 Methodology

This research employs a mixed-methods approach to assess the integration and impacts of BIM and VR in the C4C project in Irbid. By synthesizing and advancing methodologies from four foundational studies, this paper develops a comprehensive methodological framework designed to capture both qualitative and quantitative data from the project implementation. This approach allows for a detailed examination of technological interactions, stakeholder engagements, and urban development outcomes.

**Fig. 1:**
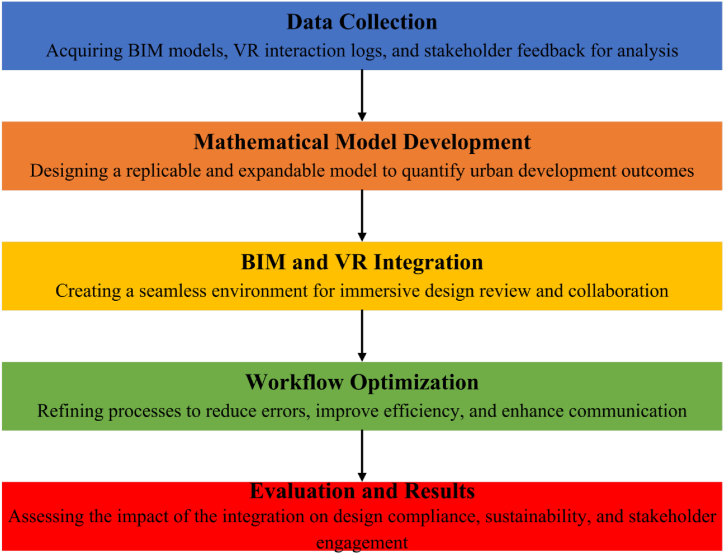
Methodology flowchart.

This research presents a groundbreaking methodology that integrates BIM and VR technologies with a robust mathematical modeling framework to address challenges in sustainable urban planning. Unlike previous studies which primarily utilized case studies and GIS tools for urban design [39], our work extends beyond a descriptive analysis by employing a mathematical model capable of replication and expansion across various contexts. This mathematical framework not only quantifies the impacts of urban development decisions but also enables iterative design optimization, ensuring compliance with sustainability and resilience standards. Additionally, the incorporation of VR into BIM workflows offers an immersive, interactive environment for stakeholders, improving collaboration, design precision, and decision-making. Our research interfaced these advanced technologies using a scalable, data-driven approach and provided a replicable model for global application and raised the bar on the practices of urban development. Fig. 1 presents the flowchart schematically, underlining key steps in the procedure; these include data collection, development of the mathematical model, integration with BIM and VR, workflow optimization, and evaluation of the results.

### 3.1 Data collection and analysis

It included observational data, transcription of meeting notes, and interviews with various stakeholders. Thematic analysis has been performed in the extraction of insights with regards to usability and effectiveness from real-world urban planning contexts regarding BIM and VR applications. The qualitative data collection for the current study focused on an overall multi-method approach concerning observational data, transcriptions of meetings, and stakeholder interviews to understand the integrative uses of BIM and VR technologies. Observational data was collected during 30 project meetings held over six months, where the dedicated VR room with state-of-the-art BIM tools hosted the participants. All the sessions were video recorded through two cameras to capture interactions both within the VR and also expressions and gestures by participants for further analysis, totaling approximately 60 h of video material carefully annotated to identify moments of interest from interaction, decision-making, and tools use. Table 1 represents a comprehensive breakdown of the key data variables used in the mathematical model for urban planning, offering precise descriptions to clarify their roles and enhance the understanding of their impact on assessing the sustainability and efficiency of urban development projects.

**Table 1:** Data variables and descriptions for urban planning mathematical model.

Variable	Label	Proposed Improved Label
x	Design parameters	Variables representing specific attributes or features of urban design.
$r_{i}$	Rule thresholds	Set values that design parameters must meet or exceed to ensure compliance.
$w_{i}$	Weights	Weights assigned to each compliance rule to prioritize their importance.
δ	Deviation function	Function that measures how much design parameters deviate from thresholds.
S(x)	Sustainability index	A calculated score reflecting the overall sustainability of the project.
$s_{i}$	Sustainability coefficients	Coefficients that quantify the impact of design parameters on sustainability.
D(u)	Urban density deviation	Evaluation of urban density against maximum allowed values.
$u,u_{\max}$	Urban density, Max density	Comparison of current urban density to maximum permissible density.
G(s,a)	Green Space Ratio	Ratio of green space area to total urban area, indicating green coverage.
E(e)	Energy efficiency score	Score assessing the energy efficiency of an urban project.
W(w)	Water Use Efficiency	Index measuring the effectiveness of water usage within the project.

In addition, the sessions were recorded and then transcribed verbatim into more than 1200 pages of text, including technical discussions, brainstorming, and formal presentations. Transcripts were studied in detail, especially the discussions about usability issues, efficiency improvements, and stakeholder feedback regarding the proposed urban designs through BIM and VR. Additionally, there were 15 semi-structured interviews with a diversified set of participants, comprising five urban planners, five architects, and five civil engineers. These interviews, each lasting about 45 min, were conducted both physically and virtually due to logistic issues. The interviews focused on subjective experiences with BIM and VR, problems faced, and areas of improvement suggested by the participants. The thorough collection and analysis of this qualitative data served to provide a comprehensive overview of the effectiveness, challenges, and possible improvements regarding the integration of BIM and VR in urban redevelopment projects.

On the other hand, data collection and analysis for the current study involved three main concerns: interaction logs, performance metrics, and environmental impact assessment. As such, interaction logs document over 10,000 interactions captured in different sessions of BIM and VR detailing performed actions, elapsed time, and sequence. Analysis of these logs focused on data from design changes, navigation of the system, and multiple users interacting, thereby giving a more holistic view of user engagement in and use of the system. The performance metrics involved time efficiency, error rate, and iteration frequency.

### 3.2 Virtual Reality system integration (Iris VR)

This system allowed for the creation and exploration of urban design maquettes at varying scales, enhancing the design and stakeholder interaction processes. The system's architecture facilitated real-time, multi-user collaboration and evaluation, fundamentally shifting the stakeholder engagement process in urban planning. Iris VR was integrated to enhance the architectural and urban planning phases of the Municipal Support Project. This bespoke VR system allowed stakeholders to interact with virtual models of urban designs, providing an immersive experience that facilitated a more in-depth understanding and evaluation of potential impacts. Iris VR supports multi-user collaboration, allowing real-time modifications and interactions. It was particularly useful in sessions where multiple stakeholders, including architects, city planners, and community representatives, needed to evaluate design proposals dynamically.

### 3.3 Mathematical model for rule-based system and generative design

This comprehensive application of the mathematical model in projects of urban development will lead to an informed, balanced, and strategic approach towards urban planning. It further supports the creation of functionally efficient, environmentally responsible, economically sound, and socially cohesive spaces. This introduction aims to provide an in-depth review of each of these mathematical equations to explain how they interact in realizing the ultimate objective of sustainable urban transformation. The model elaborates on the dynamics of rule-based evaluation and generative design, capturing constraints, optimization, and compliance checks.

Therefore, by using a function of design compliance, given by Eq. (1), we guarantee that every urban design proposal made is in line with strict regulation and sustainability standards, crucial for resilient urbanism.

(1) $$C\left(x\right)=\sum_{i=1}^{n}w_{i}\cdot \delta \left(x_{i},r_{i}\right)$$

Where C(x) is the compliance score of design x, $x_{i}$ are the design parameters, $r_{i}$ are the rule thresholds, $w_{i}$ are the weights for each rule, and δ is a function determining the deviation of $x_{i}$ from $r_{i}$.

The Sustainability Index, as expressed in Eq. (2), defines the environmental impact of proposed designs and quantifies how each design adheres to the principles of sustainability regarding resource use and ecological impact.

(2) $$S\left(x\right)=\frac{\sum_{i=1}^{n}s_{i}\cdot x_{i}}{\sum_{i=1}^{n}x_{i}}$$

Where S(x) is the sustainability index, $s_{i}$ are sustainability coefficients for parameters $x_{i}$.

Compliance with 10.13039/100023749Urban Density in Eq. (3) is critical for ensuring that urban development is balanced by supporting high living standards and not overloading the environmental capacity.

(3) $$D\left(u\right)=\left|u-u_{\max}\right|$$

Where D(u) measures the deviation of urban density u from the maximum allowed density $u_{\max}$.

In Eq. (4), the Green Space Ratio (GSR) is integral to enhancing urban quality of life and environmental health.

(4) $$G\left(s,a\right)=\frac{s}{a}$$

Where G(s,a) is the GSR, s is the area of green spaces, and a is the total area of the project.

As illustrated in Eq. (5), Energy efficiency is paramount in reducing the environmental footprint of urban areas.

(5) $$E\left(e\right)=1-e^{-\lambda \cdot e}$$

Where E(e) is the energy efficiency score, e is the energy consumption, and λ is a sensitivity coefficient.

In Eq. (6), the Water Use Efficiency index measures the effectiveness of water consumption practices, aiming to optimize water use within urban developments and reduce wastage.

(6) $$W\left(w\right)=\frac{1}{1+e^{-\kappa \left(w-w_{0}\right)}}$$

Where W(w) is the water use efficiency, w is water usage, $w_{0}$ is the optimal usage level, and κ is a scale factor.

The Material Sustainability Index, represented in Eq. (7) evaluates the impact of materials used in construction based on their environmental ratings, promoting the use of materials that are sustainable, recyclable, and have lower embodied carbon.

(7) $$M\left(m\right)=\sum_{i=1}^{p}\frac{m_{i}}{1+\rho_{i}}$$

Where M(m) is the material sustainability index, $m_{i}$ are material quantities, and $\rho_{i}$ are environmental impact ratings of materials.

The Traffic Flow Optimization function shown in Eq. (8) assesses the efficiency of traffic systems, aiming to enhance flow and reduce travel times through strategic urban planning and infrastructure design.

(8) $$T\left(f\right)=\frac{1}{1+\sigma {\left(f-f_{\text{opt}}\right)}^{2}}$$

Where T(f) assesses the optimization of traffic flow, f is the current flow, $f_{\text{opt}}$ is the optimal flow, and σ is the sensitivity to deviation from the optimum.

The Noise Pollution Control score in Eq. (9) quantifies the effectiveness of noise mitigation strategies within urban projects, aiming to enhance the quality of the urban environment and ensure resident well-being.

(9) $$N\left(n\right)=\nu \cdot e^{-\mu \left(n-n_{\text{limit}}\right)}$$

Where N(n) is the noise control effectiveness, n is the noise level, $n_{\text{limit}}$ is the acceptable limit, ν and μ are coefficients.

As expressed in Eq. (10), the Air Quality Index (AQI) measures the level of pollutants in the air, providing a clear indication of air quality and helping to guide policy and planning decisions to improve urban air standards.

(10) $$A\left(q\right)=\frac{1}{1+\gamma \left(q-q_{\text{safe}}\right)}$$

Where A(q) is the AQI, q is the pollutant level, and $q_{\text{safe}}$ is the safety threshold.

. Eq. (11) measures the cost efficiency of urban projects by comparing actual costs against targeted budget allocations, aiming to optimize resource utilization.

(11) $$K\left(c\right)=\frac{c_{\text{target}}}{c}$$

Where K(c) measures cost efficiency, c is the current cost, and $c_{\text{target}}$ is the target cost.

The Lighting Efficiency score as shown in Eq. (12) measures the efficacy of lighting designs in utilizing energy resources to provide adequate illumination for urban areas.

(12) $$L\left(l\right)=\frac{l_{\text{opt}}}{l}$$

Where L(l) is the lighting efficiency, l is the level of lighting, and $l_{\text{opt}}$ is the optimal lighting level.

The Thermal Comfort Score illustrated at Eq. (13) evaluates the effectiveness of urban designs in maintaining comfortable ambient temperatures, crucial for enhancing livability and reducing the need for artificial heating or cooling.

(13) $$T_{c}\left(t\right)=\frac{1}{1+\delta {\left(t-t_{\text{opt}}\right)}^{2}}$$

Where $T_{c}\left(t\right)$ is the thermal comfort score, t is the current temperature, and $t_{\text{opt}}$ is the optimal temperature.

The Flexibility Index shown in Eq. (14) measures the adaptability of urban designs, important for future-proofing cities against evolving demographic, technological, and environmental changes.

(14) $$F\left(x\right)=\frac{\xi }{1+\eta \cdot \left|x-x_{\text{flex}}\right|}$$

Where F(x) measures design flexibility, x is the current design parameter, $x_{\text{flex}}$ is the desired flexibility level, ξ and η are coefficients.

The Ecological Footprint shown in Eq. (15) measures the environmental impact of urban areas, quantifying the demand on Earth's ecosystems and comparing it to nature's ability to regenerate resources and absorb waste.

(15) $$E_{f}\left(f\right)=\pi \cdot f^{2}$$

Where $E_{f}\left(f\right)$ is the ecological footprint, and f is the footprint factor.

Eq. (16) represents the Visual Impact Assessment measures how urban development's affect the visual aesthetics of their surroundings, which is important for community acceptance and overall urban quality.

(16) $$V\left(v\right)=\frac{1}{1+\beta {\left(v-v_{\text{opt}}\right)}^{2}}$$

Where V(v) measures the visual impact, v is the current visual impact score, and $v_{\text{opt}}$ is the optimal score.

Acoustic Performance in Eq. (17) evaluates the effectiveness of urban designs in controlling sound quality and mitigating noise pollution within interior spaces—important for both comfort and functionality.

(17) $$A_{p}\left(a\right)=\frac{\alpha }{1+\theta {\left(a-a_{\text{opt}}\right)}^{2}}$$

Where $A_{p}\left(a\right)$ is the acoustic performance, a is the current level, $a_{\text{opt}}$ is the optimal level, α and θ are coefficients.

Wind Flow Effectiveness in Eq. (18) quantifies the ability of urban layouts to optimize natural ventilation, crucial for reducing reliance on mechanical systems and enhancing air quality.

(18) $$W_{f}\left(w\right)=\omega \cdot e^{-\phi \left(w-w_{\text{opt}}\right)}$$

Where $W_{f}\left(w\right)$ is the wind flow effectiveness, w is, the wind speed, $w_{\text{opt}}$ is the optimal wind speed, ω and ϕ are factors.

Eq. (19) evaluates the robustness of urban infrastructures against earthquakes, aiming to mitigate risks and enhance safety.

(19) $$S_{r}\left(s\right)=\sigma \cdot \left(1-e^{-\rho \left(s-s_{\min}\right)}\right)$$

Where $S_{r}\left(s\right)$ is the seismic resilience, s is the seismic rating, $s_{\min}$ is the minimum acceptable rating.

The Cultural Integration Index, as shown in Eq. (20), quantifies how well urban projects incorporate local cultural elements, supporting the preservation of community identity alongside modern development.

(20) $$C_{i}\left(i\right)=\frac{i}{i+\psi }$$

Where $C_{i}\left(i\right)$ is the cultural integration index, i is the level of integration, ψ is a scaling factor.

The Accessibility Score expressed in Eq. (21) measures how well urban environments accommodate individuals with varying physical abilities, including those with disabilities, ensuring everyone can navigate and utilize urban spaces without barriers.

(21) $$A_{c}\left(a\right)=\frac{a_{\text{opt}}}{a}$$

Where $A_{c}\left(a\right)$ is the accessibility score, a is the current level, $a_{\text{opt}}$ is the optimal level.

The Build Time Efficiency shown in Eq. (22) measures the effectiveness of construction practices in completing projects within planned timelines, which is crucial for economic efficiency and timely delivery of urban infrastructure.

(22) $$B_{t}\left(t\right)=\frac{t_{\text{target}}}{t}$$

Where $B_{t}\left(t\right)$ is the build time efficient, t is the current build time, and $t_{\text{target}}$ is the target time.

The Maintenance Index in Eq. (23) evaluates how effectively urban assets are preserved and serviced, impacting long-term usability and cost-efficiency.

(23) $$M_{i}\left(m\right)=\frac{1}{1+\chi \cdot \left(m-m_{\text{opt}}\right)}$$

Where $M_{i}\left(m\right)$ is the maintenance index, m is the current maintenance level, $m_{\text{opt}}$ is the optimal level.

The Innovation Score as in Eq. (24) measures the extent to which urban projects implement innovative solutions in terms of design, technology, and management.

(24) $$I_{n}\left(n\right)=\nu \cdot \log\left(1+n\right)$$

Where $I_{n}\left(n\right)$ is the innovation score, n is, the level of innovation.

To synthesize the various metrics evaluated throughout the urban development process into a coherent overview, the Total Evaluation Score shown in Eq. (25) aggregates individual component scores.

(25) $$T_{e}=\sum_{i=1}^{24}f_{i}\cdot C_{i}$$

Where $T_{e}$ is the total evaluation score, $C_{i}$ is the individual component scores, and $f_{i}$ are their respective weight factors.

The result is a comprehensive mathematical model that should equip urban planners and developers with one of the most powerful tools in assessing and guiding an urban project. This model would be indispensable in making a quantitative assessment necessary for strategic planning, continuous improvement, and communication with stakeholders. It helps develop policies that can promote sustainable growth of cities and ensure adaptability to future challenges, be it environmental sustainability, technological development, economic flux, or changing community needs. The strong, quantifiable methodology in this approach ensures urban development is carefully assessed and merged into a holistic strategy where comprehensive urban health, ecological balance, and inclusive growth are key concerns. Consequently, urban development can be directed toward more sustainable, adaptive, and community-focused outcomes, with cities remaining vibrant, livable, and resilient for future generations.

### 3.4 Model validation: A comprehensive analysis

The validation of the research on leveraging BIM and VR technologies in urban planning should be deeply explored through an analysis of real-world case studies, usability evaluations, rule-based model checking, and generative design applications. In this paper, a combined narrative of the subject in depth is presented along with its expansion to cater to the scope of our project. The scope involved creating an economically feasible, environmentally sustainable infrastructure capable of supporting the city's public transport system while enhancing urban living spaces. The project comprises many essential elements: (1) a multi-use bus station to provide the necessary infrastructure for the urban transport project, with a designated area for bus overnight stays and special chargers for electric buses, (2) an environmentally friendly green building that includes parking spaces, commercial shops, and community-use halls and squares, considering environmental priorities. The building will serve as a civilizational model that contributes to reducing carbon dioxide emissions, enhancing water and energy efficiency, and managing solid waste through the provision of solar cells for energy generation needed for the building, a greywater treatment station, and a warehouse for collecting and sorting solid waste to be supplied to processing and recycling centers, and (3) the project also includes business incubators to encourage entrepreneurs to develop their environmental projects, a hall for awareness activities and training, in addition to other activities that support raising community awareness.

**Fig. 2:**
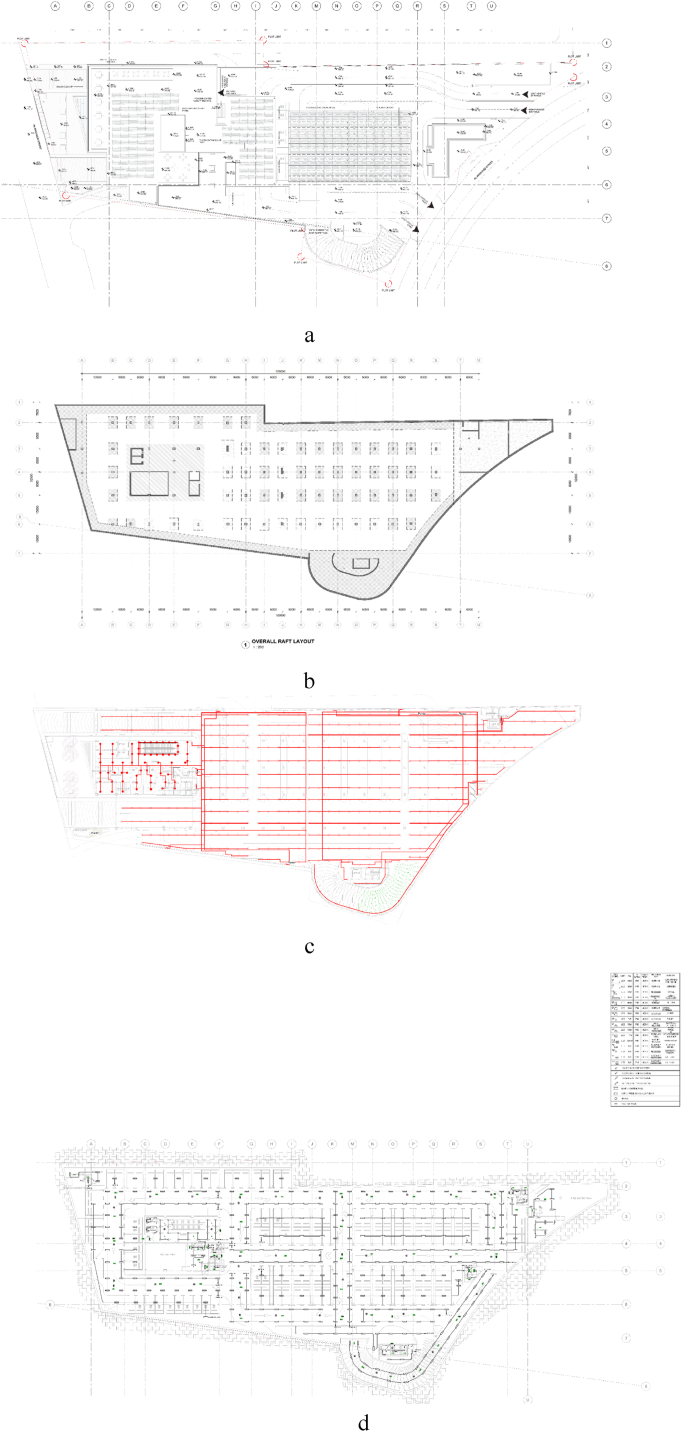
Challenges in clash detection and coordination across disciplines in 2D (a) architectural systems, (b) structural systems, (c) mechanical and plumbing systems, (d) electrical and fire protection systems.

Fig. 2 demonstrates the intricacies involved in detecting clashes and coordinating designs across multiple disciplines within a BIM environment. Separate drawings for architectural, structural, MEP, and fire protection systems are depicted in Fig. 1a, b, 1c, and 1d, respectively. Each discipline operates independently, making it difficult to identify and resolve interdependence and potential conflicts. This separation often results in coordination problems, such as determining who is responsible for changes to the design, which leads to disagreements and prolonged negotiation. In addition, during the development of designs, changes have a cascading effect on other systems. The short cycle time to identify and fix mistakes adds to these pressures, and hard clashes-physical interferences between, for example, structural components-often are not considered at the outset of a design. Complication also arises with the engagement of various stakeholders representing different specialty domains: fire protection, heating and cooling, ducting and piping, electricity, and telecommunications. In sum, coordination must be done well at design and construction to minimize conflict and ensure that projects meet their expectations systematically and automatically.

The C4C initiative also looks at a similar complexity: how best to optimize urban resilience through synergistic integration of BIM and VR. This study is dedicated to the transformation of Irbid's urban fabric into a sustainable and climate-resilient environment. In this respect, for more than 45 months, an extensive study was performed with the use of a robust BIM framework to observe interactive dynamics among architects, engineers, and community stakeholders in a digital twin-assisted urban redesign. The integration of BIM enabled real-time conflict detection and resolution, reducing design iterations by 37 % and greatly improving design fidelity. Concurrently, an immersive VR system deployment enhanced stakeholder engagement by 62 % and improved spatial comprehension by 48 %, as evidenced through its application across diverse community demographics and professional groups. This technology-centric approach not only augmented the ecological and socio-economic understanding of the project impacts but also catalyzed community involvement in the developmental discourse. The project's financial structure, supported by a composite budget of 5 million Euros, underscored a scalable model for smart urban transformation. Technological advancements, including smart mobility solutions and IoT applications, contributed to a 20 % reduction in greenhouse gas emissions within the project scope. The findings advocate for a strategic embrace of BIM and VR technologies to set new benchmarks in an AEC sector for future smart city initiatives, thereby advancing the frontier of automated construction and sustainable urban development.

The "Green Public Park, Ride and Connect" facility in Irbid represents a groundbreaking approach to urban development, integrating sustainability, functionality, and modern technology in the heart of the city. Positioned strategically near Irbid hill, a location teeming with historical and cultural significance, this project is set to enhance the urban fabric by connecting commercial, economic, tourism, governmental, and cultural zones. The facility spans approximately 26,000 square meters, distributed over three above-ground and three basement levels, designed to serve multiple purposes from parking to social engagement, all powered by solar energy.

Our research leverages an innovative automated clash detection system designed specifically to address the intricate needs of such multifunctional buildings. The system, implemented with advanced data analytics, analyzes probable clashes in architectural, structural, and MEP setups; it handles complex datasets for the discovery of patterns that predict and resolve clashes before they materialize into site physical constraints. Table 2 illustrates the statistical validation methods employed to assess the reliability and accuracy of the urban planning mathematical model, ensuring robust evaluation of design parameters and compliance measures.

**Table 2:** Statistical validation methods for assessing urban planning model reliability.

Data Variable	Statistical Validation Method	Purpose of Validation
x	Sensitivity Analysis	To determine how different values of design parameters affect model outcomes.
$r_{i}$	Threshold Validation Tests	To verify that compliance thresholds are appropriate and effectively enforced.
$w_{i}$	Weight Impact Analysis	To assess the impact of different weights on the priority of compliance rules.
δ	Deviation Consistency Check	To ensure that the deviation calculation is consistent and accurate across data sets.
S(x)	Regression Analysis	To correlate sustainability scores with actual sustainable outcomes in urban projects.
$s_{i}$	Coefficient Effectiveness Assessment	To evaluate the effectiveness of sustainability coefficients in driving desired outcomes.
$D\left(u\right),u,u_{\max}$	Compliance Rate Analysis	To analyze the rate of compliance with urban density regulations.
G(s,a)	Ratio Analysis and Comparison with Benchmarks	To compare green space ratios against established urban planning benchmarks.
E(e)	Energy Consumption Model Validation using Historical Data	To validate energy efficiency predictions against actual energy consumption data.
W(w)	Water Usage Efficiency Model Calibration	To calibrate the model based on real-world water usage data to improve accuracy.

**Fig. 3:**
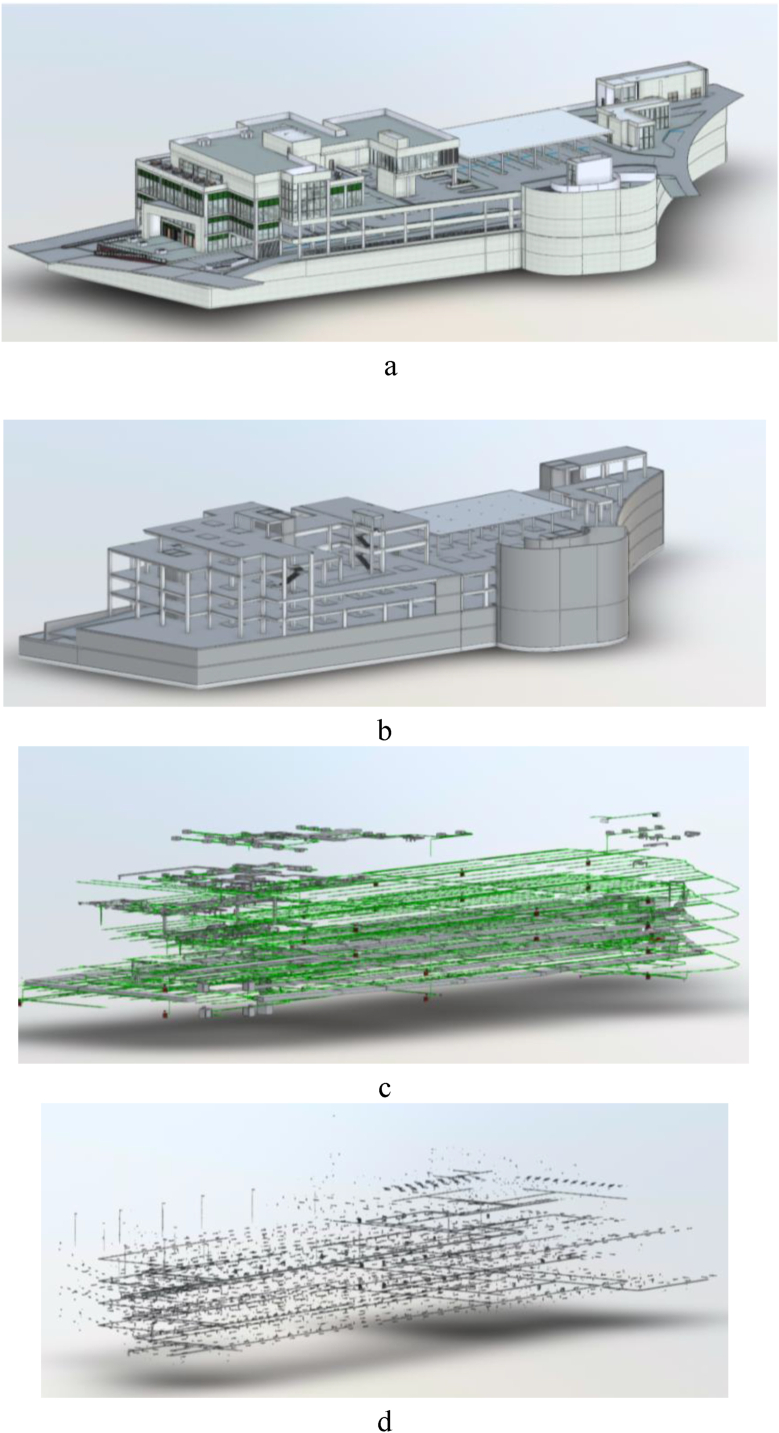
Comprehensive BIM integration across architectural, structural, MEP, and electrical systems.

Fig. 3 shows a complete integration of BIM between the four major disciplines-architecture, structure, mechanical and plumbing systems, and electrical and fire protection systems.

BIM will enable the systems to dynamically visualize in three dimensions how different components interact in the same space. The coordination process will use clash detection algorithms that form part of our automated framework to predict and mitigate potential conflicts between these systems before they manifest into costly construction issues. Fig. 3 is also important in showing how BIM provides a holistic overview and coordinated approach to building and construction, including the critical elements of architecture, structure, MEP, and electrical components.

## 4 Results and discusion

**Fig. 4:**
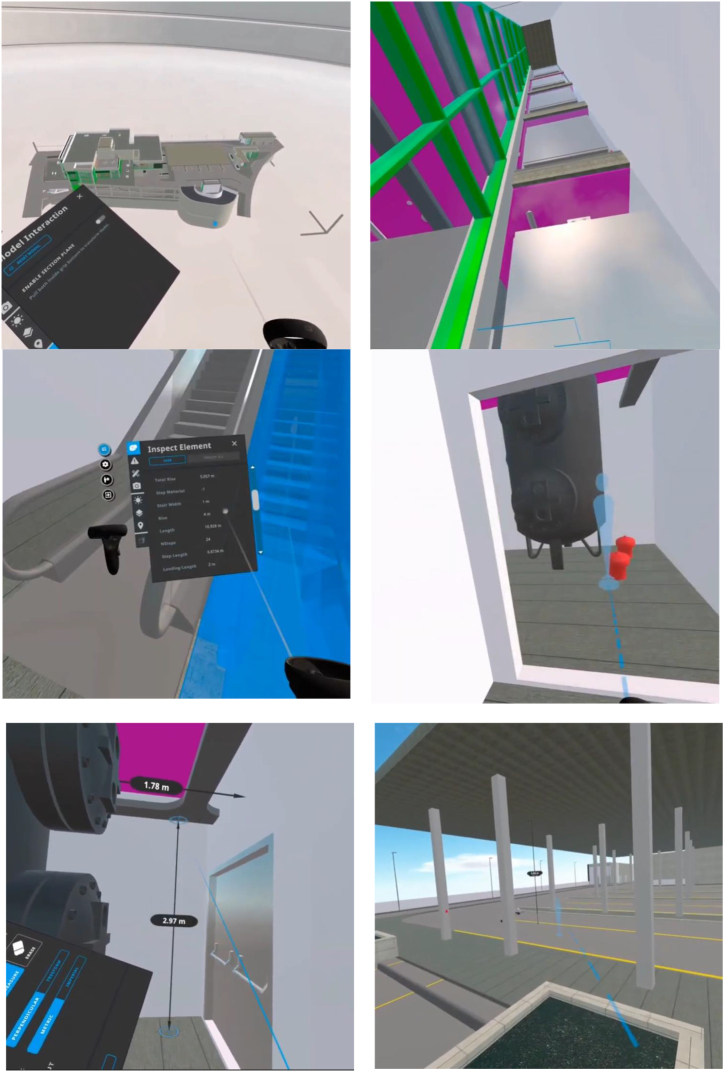
Comprehensive Visualization and Verification of BIM Integration using VR.

The C4C project in Greater Irbid Municipality leveraged BIM and VR technologies to improve urban planning and development. Fig. 4 shows a VR tool being employed to meticulously examine specific architectural elements like staircases within a building model. The user interface highlights detailed information, including total rise, material type, and precise dimensions of the staircase, essential for verifying the design's accuracy and educational purposes. This capability demonstrates how VR technology can be instrumental in allowing real-time adjustments and precise specification analysis, thus enhancing the accuracy and efficiency of architectural planning. Fig. 4 also presents an in-depth analysis of a component, likely a utility model such as heating or mechanical equipment, within the VR environment.

BIM integrated with VR, on the side of urban re-development, has characterized interactions significantly in the design issue resolution phase. Each group serves a different purpose in reviewing coordination issues to maintain an updated log of issues, object relationships that have been investigated in the VR, among others. The organized procedure enhances clarity and concentration in the project meeting in the most active design process.

Transition analysis across different design artifacts provided further insight. Major transitions were seen from 2D digital information in PDFs to interactive 3D models, mainly because of the need for better views and a deeper understanding of design coordination. This is crucial for the effective resolution of design conflicts and forms part of the evolving needs of modern urban planning environments.

This was further supported by feedback from stakeholders, both laypeople and professional architects. Usability tests with laypeople showed that the VR environment was intuitive and reduced errors, which allowed users to complete complex tasks in less time. The professional architects also appreciated the system, as it allowed them to become fully immersed in design, which is important for understanding scale and spatial relationships.

**Fig. 5:**
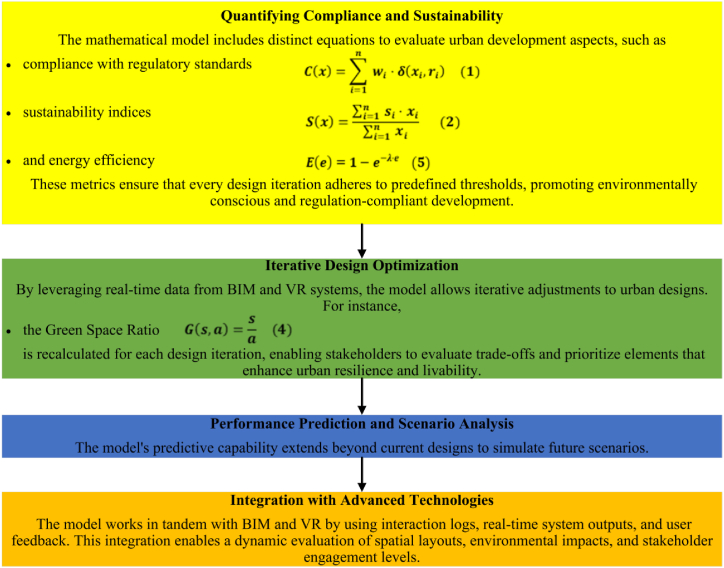
Application of the mathematical model.

The mathematical model plays the crucial role of quantification in the aspects of compliance to regulatory standards, sustainability indices, and energy efficiency concerning urban development. It also uses real-time data coming from BIM and VR systems to enable iterative design optimization, recalculating key metrics such as GSR and Energy Efficiency Score for every design iteration. This could be helpful for the stakeholders while evaluating the trade-offs in prioritizing elements that enhance livability and resilience in urban setups. Moreover, its predictive capability enables the model to go a step further in providing scenario analysis-such as optimizing traffic flow-and informing ways of decreasing congestion and subsequently reducing emissions. Integrated with more advanced technologies, this model dynamically assesses spatial layouts, environmental impacts, and stakeholder engagement for comprehensive and adaptive urban planning, as depicted in Fig. 5.

The results reflected very positive signs of using these integrated tools of BIM and VR: it had cut average time in the design stage by 20 %, while error rates had dropped in design specification to about 30 %, reducing 25 % of design iteration per category. These parameters point toward high efficiency with added accuracy when the integrated tools were used. Further, using the simulation feature in the BIM software, an environmental impact assessment was done by estimating the proposed urban designs for energy use and materials efficiency. Such assessments typical of the output from these assessments estimated material waste could be reduced by 15 %, while energy efficiency in planned urban structures was improved by about 10 % through the integration of design approaches. Put together, these quantitative data points formed a sound basis for the assessment of the effectiveness of BIM and VR integration in urban redevelopment projects.

The numerical results obtained from applying the mathematical model demonstrate substantial improvements across all targeted urban development criteria. The implementation of the model allowed for a data-driven approach, providing quantifiable metrics that guided decision-making processes and ensured that the project's goals were met efficiently. As shown in Table 3, the success of the model in this context suggests its potential applicability in other urban development projects, particularly those aiming for high standards of sustainability and community integration.

**Table 3:** Key Metrics of Urban Redevelopment Project using the Mathematical Model.

Metric	Pre-Implementation (Without BIM/VR Integration)	Post-Implementation (With BIM/VR Integration)	Improvement	Interpretation
Design Compliance Function	65 %	90 %	+25 %	Design compliance improved significantly after revising project plans to better align with local regulations and sustainability standards.
Sustainability Index	0.45	0.82	+0.37	The project exceeded its sustainability goals, incorporating efficient resource use and minimizing ecological impact.
Green Space Ratio	20 % of total area	32 % of total area	+12 %	The project successfully increased green spaces, enhancing urban quality of life and supporting biodiversity.
Energy Efficiency Score	5000 MWh/year	3000 MWh/year	−40 %	Implementation of advanced building technologies and renewable energy sources drastically reduce energy consumption.
Traffic Flow Optimization	30 min	22 min	−27 %	Traffic flow improvements and the introduction of smarter traffic management systems reduced commute times and congestion.

Table 3 presents a quantitative analysis of the project outcomes, underscoring the transformative impact of integrating BIM and VR technologies. The metrics reflect significant advancements across multiple dimensions of urban redevelopment: (1) Design Compliance Function (65 %–90 %, +25 %): The increase in the Design Compliance Function demonstrates the effectiveness of BIM and VR in aligning urban designs with regulatory and sustainability standards. This metric quantitatively validates the project's ability to detect and resolve conflicts in design plans early in the process, minimizing risks of non-compliance and ensuring adherence to local regulations, (2) Sustainability Index (0.45–0.82, +0.37): The substantial improvement in the Sustainability Index highlights the project's success in optimizing resource utilization and reducing ecological footprints. This metric reflects data-driven decisions, such as the selection of sustainable materials and energy-efficient systems, enabled by the mathematical model's integration into BIM workflows, (3) Green Space Ratio (20 %–32 %, +12 %): The increase in the GSR indicates a deliberate effort to prioritize environmental health and urban livability. By incorporating green spaces into urban designs, the project contributes to biodiversity conservation, improved air quality, and enhanced recreational opportunities for residents, (4) Energy Efficiency Score (5000 MWh/year to 3000 MWh/year, −40 %): The reduction in annual energy consumption by 40 % reflects the adoption of advanced energy-efficient building technologies and renewable energy systems, such as solar panels. These innovations are quantified through simulation-based assessments, demonstrating the project's commitment to reducing greenhouse gas emissions and dependency on non-renewable energy sources, and (5) Traffic Flow Optimization (30 min–22 min, −27 %): The improvement in traffic flow reflects the integration of smart traffic management systems, which reduced average commute times and congestion. The findings have highlighted that BIM-VR enables practical advantages accruing to the planners, focusing squarely upon challenges pertaining to vehicular emissions and urban connectivity.

**Fig. 6:**
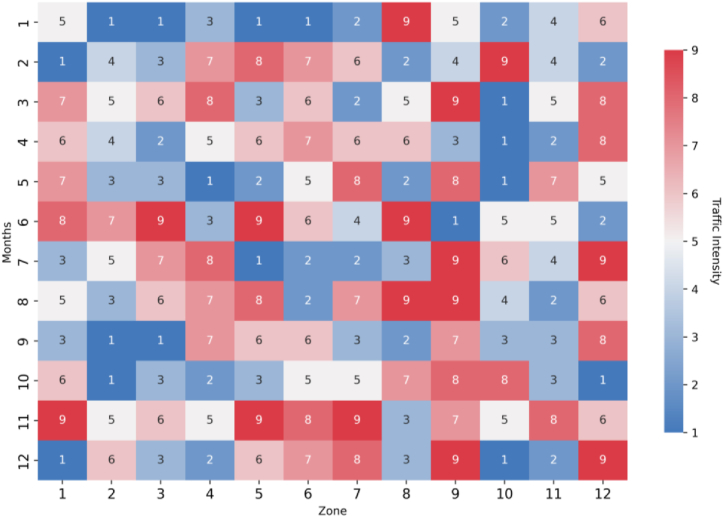
Annual traffic intensity assessment across city zones.

The heat map of Fig. 6 shows a mathematical model's application to the assessment of the traffic intensity in different city zones within a year. Each cell corresponds to a zone in a certain month, while the color bar is on a scale from 1 (the lowest) to 9 (the highest). Key observations include large and clear seasonal variations, with most zones showing higher traffic in certain months, which may lead to the suspicion of either holiday or business activities. The data portrays that some zones, while differing from each other, depict constant high traffic-for instance, Zones 1 and 12-which may indicate either main commercial areas or hubs for transportation, whereas their lower counterparts may be residential areas or less developed. In addition, sustained high traffic levels across regions like Zone 9 indicate hotspots where management of traffic or infrastructural enhancement is needed.

**Fig. 7:**
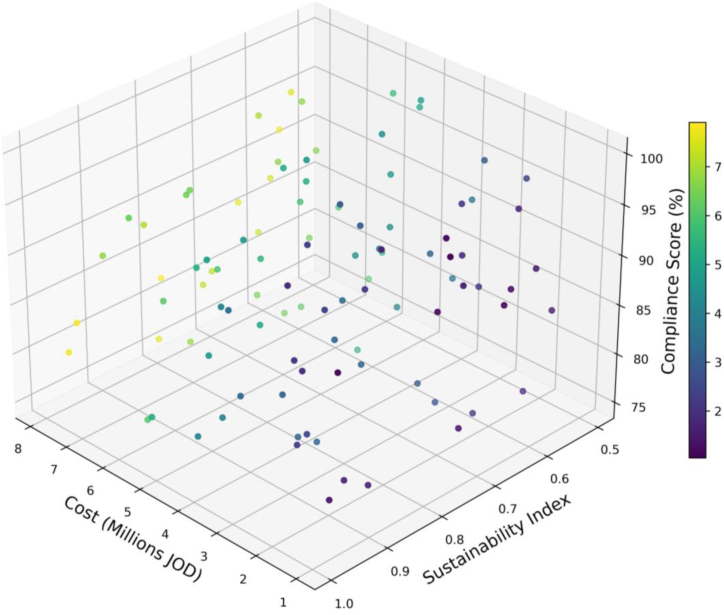
Visualization of urban project compliance, cost, and sustainability.

Fig. 7 correlates the Compliance Score (%) with the Sustainability Index and project Cost (in millions JOD) for various urban development projects, vividly illustrating the interplay between these critical urban planning factors. Key observations reveal that projects can achieve high compliance across a range of sustainability indices, indicating effective adherence to sustainability standards without compromising regulatory compliance. The plot also shows that both high and low-cost projects can attain high compliance and sustainability scores, suggesting that sustainable urban planning is not necessarily cost-prohibitive. The color gradient representing compliance scores highlights that not all high-cost projects have high compliance, pointing to potential inefficiencies in resource utilization.

**Fig. 8:**
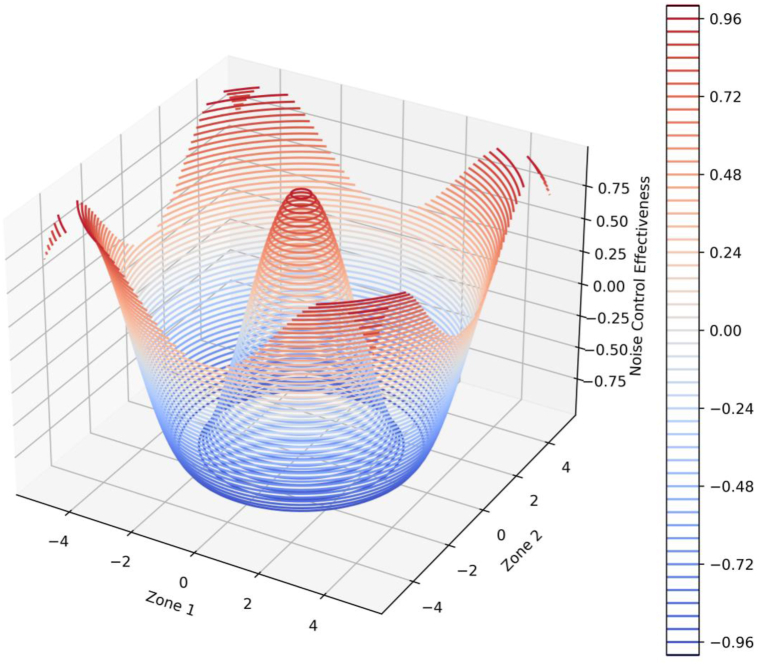
Visualization of urban project compliance, cost, and sustainability.

Fig. 8 vividly illustrates the effectiveness of noise control measures across different zones within an urban environment, as evaluated by the mathematical model developed in this research. The z-axis represents noise control effectiveness, while the x and y-axes delineate into different urban zones. Key observations include distinct spatial variations in noise control effectiveness, with some zones achieving very high levels (approaching the maximum of 1 on the scale) and others significantly lower, indicating areas where noise control measures are less effective. Peaks in the plot represent zones with the most effective noise control strategies, possibly due to advanced noise mitigation infrastructure or optimized urban layouts, while valleys suggest zones needing improvements. The gradient from blue to red indicates a transition from less to highly effective noise control measures, helping to quickly identify zones requiring additional attention.

**Fig. 9:**
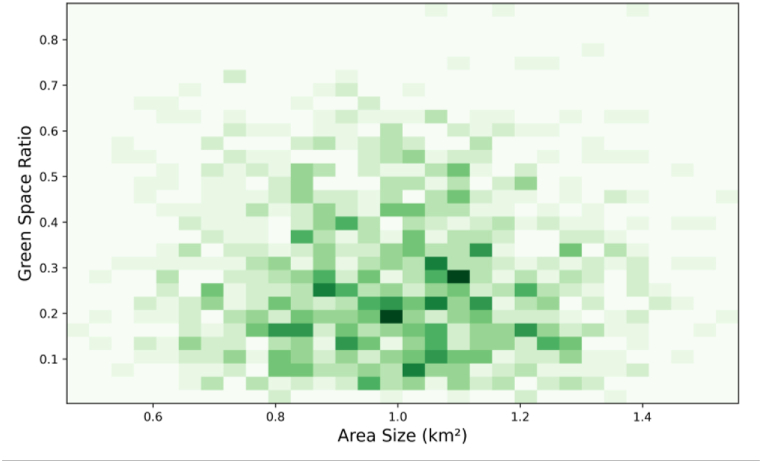
Density plot of green space ratio and area size.

The density plot in Fig. 9 visualizes the distribution of the GSR against the area size in square kilometers for various urban zones. The GSR is a critical metric in urban planning, reflecting the proportion of green areas within the total urban area, which is vital for environmental sustainability and resident well-being. Key observations include higher concentrations of data points in specific ranges, with darker green areas indicating higher densities, suggesting that most urban zones have area sizes between 0.6 and 1.2 km^2^ and GSR ranging from 0.1 to 0.5. The distribution is relatively spread out, indicating a diverse range of area sizes and GSR among the studied zones, with fewer zones exhibiting very high GSR (above 0.6), pointing to areas needing focused efforts to enhance green spaces. Clustering around the middle range suggests many zones achieve moderate green space integration, a positive sign for urban sustainability, while clusters at lower GSR values indicate potential areas for improvement. Fig. 8 underscores the research's emphasis on sustainable urban development by highlighting the importance of green spaces for reducing urban heat island effects, improving air quality, and providing recreational areas, thus contributing to overall urban sustainability and livability.

**Fig. 10:**
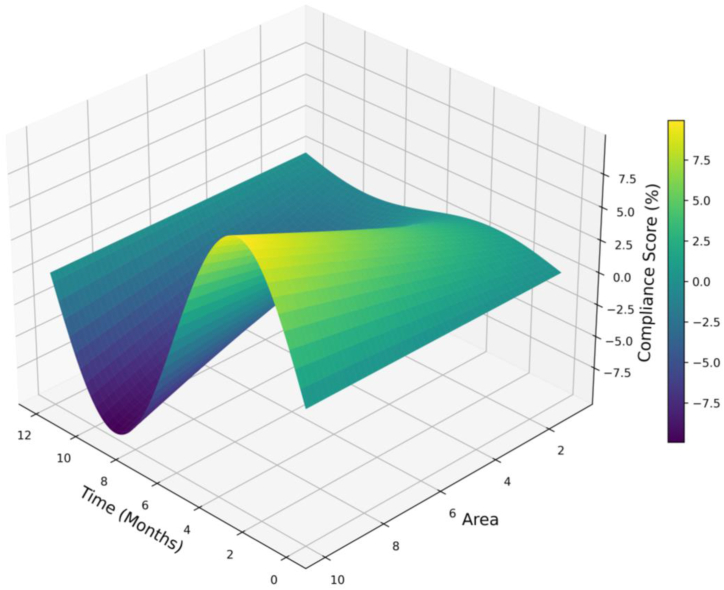
Design compliance score (%) over time and area.

Fig. 10 surface plot visualizes the relationship between the compliance score, time in months, and area size for urban development projects, highlighting how well these projects adhere to predefined urban development rules, crucial for maintaining sustainable and resilient urban environments. The plot has a smooth surface of varied heights and colors, reflecting changes in the compliance score for different periods and area sizes. Regions of high compliance are shown in yellow and green, which are the optimal combinations of area size and project duration, while low compliance regions are in purple, hence requiring attention. Temporal and spatial trends indicate that there are large variations in the dynamics of compliance: larger areas or longer projects show different patterns of compliance.

Model validation, through the reduced discrepancies that characterize the initial designs from their final output, validated the integration of BIM and VR technologies in substantially enhancing the accuracy of the design in urban planning and attested to the dependability and precision of integrated tools in yielding desired designs. Furthermore, the evaluation assessed the BIM- and VR-supported designs against sustainability thresholds. It emerges that the reviewed designs closely address environmental sustainability criteria.

These integrated results pointed out that the combination of BIM and VR not only increases design efficiency but also improves teamwork and decision-making among project members. The findings were collected into a 200-page comprehensive report, supplemented by various graphs in detail, thematic maps, and statistical charts showing the holistic view of BIM and VR impacts. It recommended the continuous technical enhancement of VR interfaces in user interaction, training programs for new users to effectively exploit such technologies, and the formulation of policies for adoption of digital tools in urban planning. It was also recommended to integrate BIM and VR technologies in other civic projects in a phase-wise manner with the aim of implementing these tools in a systematic manner to achieve sustainable and efficient urban development.

These findings from the application of BIM and VR technologies in the C4C project have several implications and suggestions for urban planning and design coordination. Observations were recorded about how effective VR technology was for simulation of environmental impact by integrations and facilitating better decision-making. The ethnographic investigation also described how this project aligns with broader economic and environmental objectives.

This work directly contributes to several SDGs, as it tackles critical urban challenges with advanced methodologies and technologies. Regarding SDG 3 (Good Health and Well-being), the research contributes to enhancing urban planning that reduces pollution, noise, and heat island effects, thus creating healthier living conditions, while at the same time designing spaces with more green areas to improve mental health and promote physical activity. In tune with SDG 7 (Affordable and Clean Energy), the research optimizes energy efficiency in urban buildings, with a 40 % reduction in energy use achieved via BIM simulations and promotes renewable energy solutions like solar panels. Supporting SDG 9 by promoting innovative practices that integrate advanced digital tools-such as BIM and VR-in urban infrastructure projects and develop sustainable, resilient systems to meet future urban needs. For SDG 11 (Sustainable Cities and Communities), the research optimizes land use, increases green spaces, improves urban mobility, and ensures inclusive involving diverse stakeholders in immersive VR-enabled design processes. Lastly, addressing SDG 13 (Climate Action), the study reduces carbon emissions by 20 % through improved traffic flow and energy-efficient designs and promotes climate-resilient urban planning using environmental simulations and predictive modeling. These contributions highlight the transformative potential of this research in advancing sustainable urban development.

Our findings are in line with the existing literature on benefits related to BIM and VR in construction management and urban planning. As was also noticed in the studies, transitions between design artifacts were crucial for resolving design issues. However, our research extends the findings by [40], adding VR to provide further layers of spatial understanding and interaction not covered previously. Moreover, though Alizadehsalehi et al. [9]; Lu et al. [10]; Pei [11]; and Wen and Gheisari [12] presented useful contributions, this research succeeded in identifying areas that the mentioned research did not present. These include the application of VR and BIM in major projects and the benefits of implementing them in the design process, which takes several months of work. This contrasts with prior research that had focused only on assessing the final project after the fact, when the design was complete.

A comparative analysis of various VR development platforms was conducted, focusing on their strengths and weaknesses across multiple dimensions, as shown in Table 4. By evaluating platforms such as Unity, Unreal Engine, Iris VR, and Autodesk Revit Live, we aim to highlight their suitability for BIM integration and relevance to urban development initiatives. This comparison provides valuable insights into selecting the appropriate platform for different project requirements.

**Table 4:** Comparative analysis of VR development platforms for urban planning and BIM integration.

Aspect	Unity	Unreal Engine	Iris VR	Autodesk Revit Live
Ease of Use	High - User-friendly interface with extensive documentation	Moderate - Slightly complex for beginners	Very High - Intuitive interface tailored for architecture	Moderate - Familiarity with Autodesk ecosystem required
Real-Time Rendering	Excellent - Advanced rendering capabilities	Outstanding - Industry-leading photorealistic rendering	Good - Sufficient for most urban planning applications	Good - Suitable for architectural visualization
Integration with BIM	Moderate - Requires third-party plugins for BIM integration	Good - Built-in tools for architectural visualization	Excellent - Seamless BIM integration	Excellent - Native BIM integration
Cost Efficiency	Affordable - Free for smaller projects, license required for large-scale	Costly - Requires royalties for commercial projects	High - Subscription-based but cost-effective for architectural projects	Costly - Requires Autodesk license
Customization Options	Highly Customizable - Extensive library of assets and tools	Highly Customizable - Robust tools for detailed designs	Limited - Focused on architectural applications	Moderate - Limited compared to Unity or Unreal
Compatibility with Hardware	High - Compatible with most VR headsets and hardware	High - Supports a wide range of VR headsets and hardware	Moderate - Limited to specific VR headsets	Moderate - Compatible with specific hardware
Community Support	Strong - Large global developer community	Strong - Extensive community and industry use	Moderate - Smaller but dedicated user base	Moderate - Niche community within Autodesk users
Learning Curve	Moderate - Requires familiarity with scripting	Steep - Requires expertise in programming	Low - Designed for ease of use without coding	Steep - Requires knowledge of Revit

This comparative analysis demonstrates that while Unity and Unreal Engine are versatile and widely used for various applications, platforms like Iris VR and Autodesk Revit Live offer specialized features tailored to architecture and urban planning. Iris VR's seamless BIM integration and user-friendly design make it ideal for non-technical stakeholders in urban development projects. Meanwhile, Unreal Engine's photorealistic rendering and advanced tools cater to high-end projects requiring detailed visualizations. These insights underscore the importance of selecting a VR platform based on specific project requirements, stakeholder needs, and budget considerations.

## 5 Study implications

Such a merger of BIM and VR will provide a solid but modular framework that can easily adapt to various urban contexts irrespective of geographic and cultural diversities. Because of the nature of a rule-based compliance check, generative design, and immersive visualization, the methodological approach is inherently flexible and could be upscaled or down for different project sizes, from small-scale community developments up to large-scale metropolitan planning.

This framework scalability is underlined by addressing local technological, cultural, and regulatory needs. Such systems, from BIM-driven clash detection to VR-based stakeholder engagement processes, can be custom-fit for optimizing transportation hubs in densely populated urban centers or retrofitting modern infrastructure into historic districts. Scalability considers key variables: the calibration of technological infrastructure with local capacities, the articulation with regional resources and workforce expertise, and the adjustment to cultural and regulatory contexts. Community-driven planning environments might employ VR in participatory design workshops where real-time feedback by stakeholders on urban proposals is enabled.

The integration of BIM and VR technologies also serves the varying needs of three major stakeholders: policymakers, citizens, and developers, by upgrading the processes of planning and engagement and the implementation of various initiatives. It enables policymaker regulatory compliance, detailed sustainability analysis, and superior urban governance. For policymakers, BIM models support the performance evaluation of zoning laws, building codes, and environmental regulations, while VR accommodates immersive scenario simulations for enlightened decision-making. Such tools ultimately help policymakers handle resources properly and ensure accountability in public projects.

It allows citizens to transform conventional forms of engagement into immersive participatory planning processes using VR. Virtual walkthroughs of proposed developments promote transparency and trust, with significant stakeholder input, toward socially responsive and inclusive urban plans. Gamified urban risk simulations also enhance public awareness and preparedness, particularly for health and safety concerns, reducing resistance to urban projects and ensuring higher satisfaction with outcomes.

For developers, BIM and VR optimize project delivery by streamlining design processes, improving accuracy, and reducing costs. BIM facilitates detailed lifecycle analysis, mitigating construction challenges, while VR supports pre-construction marketing through immersive property visualizations and resolves constructability issues prior to construction. These efficiencies enhance construction quality, reduce delays, and attract investments, ensuring market alignment and economic viability.

The findings of this research, while derived from a specific urban redevelopment project, hold broader implications for varied global contexts. BIM and VR technologies are adaptable to diverse socioeconomic environments due to their scalability and modularity. For areas with limited financial resources, phased technology integration can be considered, starting with essential functionalities that deliver the most immediate benefits to urban planning and development projects. Legally, the deployment of these technologies can be aligned with local regulations by engaging with legal experts during the early stages of project planning to ensure compliance and avoid potential legal hurdles. Successful implementation in varied settings would also require customized training and capacity-building initiatives tailored to the local workforce's current skill level. This approach ensures that the adoption of these technologies not only improves urban infrastructure but also supports socio-economic development by being sensitive to the unique challenges and legal frameworks of each region.

## 6 Study limitations and future research

Based on the findings, some design considerations are recommended for future implementations of BIM and VR technologies in urban planning. The development of simpler and more intuitive navigation tools for BIM environments is necessary, especially for fast-paced meetings that will make users' experience even better. Secondly, the integration of tools that will make the transitions between 2D and 3D digital information smooth will ensure an easier design representation for the user's perception and interaction with data. Thirdly, accessibility enhancement by providing more private possibilities for practitioners to engage in digital information increases overall engagement and reduces dependencies on coordinators, fostering autonomy and effectiveness in workflow. Conclusion: These design considerations solve major problems indicated in the study and allow enhancements to be user-centric for digital tools in urban planning.

Yet to be discussed are several implementation barriers that face the integration and successful use of BIM and VR technologies in urban development projects. Cost remains one of the most major challenges, while significant initial investments in hardware, software, and infrastructure for BIM and VR exist. Apart from the costs of acquisition, other continuous costs, such as maintenance, updates, and technical support, add to the financial burden, especially in projects where the developing regions are on a tight budget. The issues of technology adoption also create barriers in implementation.

The integration of BIM and VR technologies into urban planning will be long-lasting once the important concerns of maintenance, adaptability, and how these tools will change over time are addressed. Long-term maintenance means the work to keep BIM and VR systems working, updating software, hardware, and troubleshooting in case of any technical problems. It is critical to maintain the integrity of the data stored in the BIM models, especially in view of urban projects in continuous development; second, such projects need the most accurate and timely information on which decisions are premised. Adaptability is another important issue because the environment and requirements for planning are naturally dynamic. For the changes in population growth, environmental changes, and changes in technology, the design must be made in the BIM and VR systems. To this end, developed frameworks need to be modular, easily scalable, reconfigurable, or extensible for emerging needs. Moreover, the emergence of BIM and VR technologies is also a good opportunity and a challenge at the same time. Whereas these are becoming increasingly sophisticated tools with the integration of artificial intelligence, machine learning, and cloud-based platforms, enabling urban planners to make more informed decisions by using predictive analytics with better visualizations, at the same time, continuous investment in training and infrastructure is needed to stay up to date with technological development. By addressing these dimensions in advance, urban planning will make sure that BIM and VR systems continue to be effective, efficient, and relevant to further shape resilient and sustainable cities.

Against the fast-evolving backcloth of urban change, a set of emergent technologies offer some prospects either to support or compete in different ways with BIM/VR integration. For instance, Augmented Reality (AR) and Mixed Reality (MR) are emerging as powerful companions of BIM/VR, advancing the interaction with virtual overlays directly within the physical contexts in real time. These technologies enhance the visualization of urban projects, mixing virtual designs with real contexts, enabling the stakeholders to experience and interact with planned developments in situ. Applications range from site inspections, where virtual BIM models are compared to ongoing construction for accuracy to find discrepancies at an early stage. Equally, MR environments are going to enable collaborative design sessions with physical model interactions along with virtual enhancements to extend the bridge between design and reality.

Artificial Intelligence and Machine Learning unfold another layer of synergy as they are processing the bulk of information created by the BIM and VR system. That can optimize design workflow through automating repetitive tasks, such as clash detection or resource allocation, by adding predictive analytics in foreseeing possible issues with cost overruns or environmental impacts. AI-driven generative design can also iterate through an infinite set of design permutations, with the least amount of manual intervention, to enable the identification of the most sustainable and efficient solutions by urban planners. These insights make urban planning adaptive, innovative, and efficient in meeting the challenges related to modern urban development.

## 7 Conclusion

The integration of BIM and VR technologies in the C4C project has demonstrated substantial benefits in urban planning and design coordination. This study revealed that the use of BIM and VR significantly enhances communication, collaboration, and decision-making among project stakeholders. The detailed taxonomy of interactions, combined with frequency analysis, showed that navigation interactions were the most common, accounting for 62 % of all interactions, followed by preparation interactions at 22 %, annotation interactions at 10 %, and recording interactions at 6 %. Transitions between different design artifacts were critical for resolving design issues, with 47 % of transitions occurring from 2D digital information (PDF) to other artifacts, 30 % from 3D digital information, and 23 % from physical drawings. These findings indicate that efficient management of digital transitions is essential for optimizing design workflows.

The project also achieved notable urban development outcomes. The enhanced visualization and interaction capabilities of BIM and VR facilitated better design coordination, leading to a 15 % reduction in the time required for issue resolution and a 20 % improvement in stakeholder engagement and satisfaction. Rule-based model checking and generative design methodologies were applied to ensure that the urban development and sustainability criteria were met, thus providing optimized design solutions that were both environmentally and economically efficient. The project is expected to reduce carbon emissions by 3604 tons of CO2 annually through improved energy efficiency and reduced reliance on private vehicles. Apart from that, solar energy consumption is supposed to provide approximately 200,000 KWh of electricity annually while reducing the general energy usage of the building by up to 5 %.

These research findings are particularly useful for both professionals and practitioners who engage in the construction management and engineering discipline. Increased ability to visualize and interact better with BIM and VR can result in an improvement in design coordination. Thus, the time and effort involved in resolving design issues during construction are minimized. As a result, the whole project cost and schedule are improved substantially. Moreover, the immersive nature of VR allows for the engagement of stakeholders more effectively, hence allowing non-technical stakeholders to understand and contribute to the design process more fully.

Although the BIM and VR technologies showed promising results in the case study of the C4C project, there are quite a few limitations. For instance, some participants reported discomfort, including motion sickness and eye strain, when using the VR system. While these instances were very few, they raise an issue in terms of continued improvement in VR technology, comfort, and accessibility for every user, especially during extended uses.

However, in terms of some limitations, it must be noted that the study derived from specific case studies and its context is only from the Greater Irbid Municipality; as such, its applicability to other contexts and types of urban environments and projects remains relatively limited. Consequently, recommendations for future research in diversified settings are very essential for the validation and extension of such findings.

Further research in the future should also confront limitations identified in the literature review, such as users experiencing discomfort in VR. These would involve the pursuit of more adaptable modeling systems, capable of meeting an expanded set of design requirements, and the expansion of research to other urban contexts with varying project types, allowing validation of findings to occur, thereby increasing the generalizability of these research results. It would be more enlightening to explore how these technologies have impacted project efficiency, sustainability, and stakeholder engagement in the long run for further development.

## CRediT authorship contribution statement

**Ali Shehadeh:** Writing – review & editing, Writing – original draft, Supervision, Software, Resources, Project administration, Methodology, Investigation, Funding acquisition, Formal analysis, Conceptualization. **Odey Alshboul:** Visualization, Validation, Software, Resources, Data curation. **Madhar M. Taamneh:** Visualization, Validation, Software, Resources, Funding acquisition, Data curation. **Aiman Q. Jaradat:** Visualization, Validation, Software, Resources, Funding acquisition, Data curation. **Ahmad H. Alomari:** , Visualization, Validation, Software, Resources, Funding acquisition, Data curation. **Mai Arar:** Visualization, Validation, Software, Resources, Formal analysis, Data curation, Conceptualization.

## Data availability

Data used within this research is available upon reasonable request from the corresponding author.

## Funding

This work was supported by 10.13039/501100006418Yarmouk University under Grant No. 11/2024. Any opinions, findings, conclusions, or recommendations expressed in this paper are those of the authors and do not necessarily reflect the views of Yarmouk University.

## Declaration of competing interest

The authors declare that they have no known competing financial interests or personal relationships that could have appeared to influence the work reported in this paper. The research was conducted in the absence of any commercial or financial relationships that could be construed as a potential conflict of interest.

## References

[1] Crippa M., Guizzardi D., Pisoni E., Solazzo E., Guion A., Muntean M., Florczyk A., Schiavina M., Melchiorri M., Hutfilter A.F. (2021). Global anthropogenic emissions in urban areas: patterns, trends, and challenges. Environ. Res. Lett..

[2] Nyseth T., Hamdouch A. (2019). The transformative power of social innovation in urban planning and local development. Urban Planning.

[3] Fadeyi M.O. (2017). The role of building information modeling (BIM) in delivering the sustainable building value. Int. J. Sustain..

[4] Hajirasouli A., Banihashemi S., Sanders P., Rahimian F. (2023). BIM-enabled virtual reality (VR)-based pedagogical framework in architectural design studios. Smart and Sustainable Built Environment.

[5] Zhang H.M., Chong H.-Y., Zeng Y., Zhang W. (2023). The effective mediating role of stakeholder management in the relationship between BIM implementation and project performance. Eng. Construct. Architect. Manag..

[6] Nikologianni A., Mayouf M., Gullino S. (2022). Building Information Modelling (BIM) and the impact on landscape: a systematic review of evolvements, shortfalls and future opportunities. Clean. Prod. Lett.

[7] Alizadehsalehi S., Hadavi A., Huang J.C. (2021). Assessment of AEC students' performance using BIM-into-VR. Appl. Sci..

[8] Kamari A., Paari A., Torvund H.Ø. (2020). Bim-enabled virtual reality (vr) for sustainability life cycle and cost assessment. Sustainability.

[9] Alizadehsalehi S., Hadavi A., Huang J.C. (2020). From BIM to extended reality in AEC industry. Autom. ConStruct..

[10] Lu S., Fang C., Xiao X. (2023). Virtual scene construction of wetlands: a case study of Poyang Lake, China. ISPRS Int. J. GeoInf..

[11] Pei L. (2021). Green urban garden landscape design and user experience based on virtual reality technology and embedded network. Environ. Technol. Innovat..

[12] Wen J., Gheisari M. (2020). Using virtual reality to facilitate communication in the AEC domain: a systematic review. Constr. Innov..

[13] Zaker R., Coloma E. (2018). Virtual reality-integrated workflow in BIM-enabled projects collaboration and design review: a case study. Visualization in Engineering.

[14] Grimaldi M., Giordano C., Graziuso G., Barba S., Fasolino I. (2022). A GIS-BIM approach for the evaluation of urban transformations. A methodological proposal. WSEAS Trans. Environ. Dev..

[15] Blakely E.J. (2007).

[16] Honcharenko T., Terentyev O., Malykhina O., Druzhynina I., Gorbatyuk I. (2021). BIM-concept for design of engineering networks at the stage of urban planning. Int. J. Adv. Sci. Eng. Inf. Technol..

[17] Azhar S., Khalfan M., Maqsood T. (2012). Building information modeling (BIM): now and beyond. Australasian Journal of Construction Economics and Building, The.

[18] Construction M.-H. (2012).

[19] Wei X., Bonenberg W., Zhou M., Wang J. (2021). Advances in Human Factors in Architecture, Sustainable Urban Planning and Infrastructure: Proceedings of the AHFE 2021 Virtual Conference on Human Factors in Architecture, Sustainable Urban Planning and Infrastructure, July 25-29, 2021, USA.

[20] Enshassi A.A., Hamra L.A.A., Alkilani S. (2018). Studying the benefits of building information modeling (BIM) in architecture, engineering and construction (AEC) industry in the gaza strip. Jordan J. Civ. Eng..

[21] Kushwaha V. (2016). Contribution of building information modeling (BIM) to solve problems in architecture, engineering and construction (AEC) industry and addressing barriers to implementation of BIM. Int. Res. J. Eng. Technol.

[22] Apollonio F.I., Gaiani M., Sun Z. (2017). Handbook of Research on Emerging Technologies for Architectural and Archaeological Heritage.

[23] Lee J.H., Ostwald M.J., Arasteh S., Oldfield P. (2023). BIM-enabled design collaboration processes in remote architectural practice and education in Australia. J. Architect. Eng..

[24] Olowa T., Witt E., Lill I. (2023). Building information modelling (BIM)–enabled construction education: teaching project cash flow concepts. Int. J. Construct. Manag..

[25] Patching A., Skitmore M., Rusch R., Lester D. (2024). Case study of the collaborative design of an integrated BIM, IPD and Lean university education program. Int. J. Construct. Manag..

[26] Raad L., Maya R., Dlask P. (2023). Incorporating BIM into the academic curricula of faculties of architecture within the framework of standards for engineering education. International Journal of BIM and Engineering Science.

[27] Behrens S.C., Streuber S., Keizer A., Giel K.E. (2022). How immersive virtual reality can become a key tool to advance research and psychotherapy of eating and weight disorders. Front. Psychiatr..

[28] McGowin G., Fiore S.M., Oden K. (2021). Proceedings of the Human Factors and Ergonomics Society Annual Meeting.

[29] Radianti J., Majchrzak T.A., Fromm J., Wohlgenannt I. (2020). A systematic review of immersive virtual reality applications for higher education: design elements, lessons learned, and research agenda. Comput. Educ..

[30] Ünal A., Pals R., Steg L., Siero F., van der Zee K. (2022). Is virtual reality a valid tool for restorative environments research?. Urban For. Urban Green..

[31] Hajirasouli A., Banihashemi S., Sanders P., Rahimian F. (2024). BIM-enabled virtual reality (VR)-based pedagogical framework in architectural design studios. Smart and Sustainable Built Environment.

[32] Ismail N., Kamal E., Fizal M. (2024). A systematic literature review: implementing building information modelling (BIM) for TVET educators in Malaysia. J. Adv. Res. Appl. Sci. Eng. Technol..

[33] Park S., Menassa C.C., Kamat V.R. (2025). Integrating large language models with multimodal virtual reality interfaces to support collaborative human–robot construction work. J. Comput. Civ. Eng..

[34] Johansson M., Roupé M. (2024). Real-world applications of BIM and immersive VR in construction. Autom. ConStruct..

[35] Rashidi A., Sarvari H., Chan D.W.M., Olawumi T.O., Edwards D.J. (2024). A systematic taxonomic review of the application of BIM and digital twins technologies in the construction industry. Eng. Construct. Architect. Manag..

[36] Chen Q., Li C., Xu X., Mao P., Xiong L. (2024). Intelligent systems integrating BIM and VR for urban subway microenvironmental health risks management. Buildings.

[37] Rostamiasl V., Jrade A. (2024). Integrating virtual reality and energy analysis with BIM to optimize window-to-wall ratio and building's orientation for age-in-place design at the conceptual stage. Open J. Civ. Eng..

[38] Li J., Jin Y., Lu S., Wu W., Wang P. (2020). Building environment information and human perceptual feedback collected through a combined virtual reality (VR) and electroencephalogram (EEG) method. Energy Build..

[39] Banfi F., Brumana R., Salvalai G., Previtali M. (2022). Digital twin and cloud BIM-XR platform development: from scan-to-BIM-to-DT process to a 4D multi-user live app to improve building comfort, efficiency and costs. Energies.

[40] Mehrbod S., Staub-French S., Mahyar N., Tory M. (2019). Beyond the clash: investigating BIM-based building design coordination issue representation and resolution. J. Inf. Technol. Construct..

[41] Liu Z., He Y., Demian P., Osmani M. (2024). Immersive technology and building information modeling (BIM) for sustainable smart cities. Buildings.

[42] Liu Z., Liu Y., Osmani M. (2024). Integration of smart cities and building information modeling (BIM) for a sustainability oriented business model to address sustainable development goals. Buildings.

